# Interpeak intervals of cortical components (P1-N1-P2): a comparative study between typical individuals and those with tinnitus disorder

**DOI:** 10.1590/2317-1782/e20250130en

**Published:** 2026-01-30

**Authors:** Hélinton Goulart Moreira, Christine Grellmann Schumacher, João Vitor de Aguiar Barcelos, Bruna Ribas Maia, Fabiana Cristina Toillier, Larissa Coradini, Isabela Pohlmann de Ávila Lourenço, Pedro Vinícius de Godoy Ferrão, Dayane Domeneghini Didoné, Michele Vargas Garcia

**Affiliations:** 1 Programa de Pós-graduação em Distúrbios da Comunicação Humana, Departamento de Fonoaudiologia, Universidade Federal de Santa Maria – UFSM - Santa Maria (RS), Brasil.; 2 Curso de Fonoaudiologia, Universidade Federal de Santa Maria – UFSM – Santa Maria (RS), Brasil.; 3 Universidade Federal de Santa Maria – UFSM - Santa Maria (RS), Brasil.; 4 Programa de Pós-graduação em Distúrbios da Comunicação Humana, Departamento de Fonoaudiologia, Universidade Federal de Santa Maria – UFSM - Santa Maria (RS), Brasil.

**Keywords:** Tinnitus, Evoked Potentials, Adults, Central Nervous System, Auditory Cortex

## Abstract

**Purpose:**

To compare neural response times among cortical components of the Long-Latency Auditory Evoked Potential (LLAEP) by measuring latency and interpeak intervals in individuals with and without tinnitus.

**Method:**

This was an analytical, cross-sectional, and quantitative study. A total of 28 participants were included, divided into two groups: the Control Group, comprising 12 individuals without tinnitus perception, and the Study Group, comprising 16 individuals diagnosed with tinnitus disorder. Assessments were conducted over two sessions. Initially, all participants underwent a semi-structured anamnesis, basic audiological evaluation, behavioral tests of central auditory processing, as well as neuropsychological and tinnitus assessments. On the second day, verbal LLAEP and neurodiagnostic ABR were performed. The responses were compared by analyzing the latency of P1, N1, and P2 potentials. A between-group comparison was conducted using an independent sample t-test.

**Results:**

A statistically significant difference was observed in the interpeak intervals of P1-P2 potentials, as well as a trend toward significance in N1-P2 interpeak intervals between groups in the right ear. Additionally, a significant difference was found in P1-P2 interpeak intervals between groups in the left ear.

**Conclusion:**

Individuals with tinnitus disorder exhibited longer interpeak intervals, suggesting central auditory processing dysfunction and increased neural response within auditory processing pathways.

## INTRODUCTION

Initially, the evaluation and treatment of tinnitus focused on the inner ear, cochlear nerves, and auditory system, seeking to understand its pathophysiology. With the advancement of research, the neurophysiological model emerged as the main explanation for the perception and manifestations of this symptom. This model suggests that tinnitus results from increased spontaneous neural activity, even without a specific acoustic stimulus, which ends up activating different areas of the brain, with an emphasis on the limbic system^(1) .^

In recent years, scientific investigations have begun to focus on the neural networks associated with tinnitus and their interaction with other brain regions and body systems. This approach broadens the understanding of the symptom beyond its primary causes, exploring how different areas of the brain contribute to its perception. The predictive coding theory, proposed by Sedley et al.^(2)^ suggests that tinnitus can be interpreted as a spontaneous prediction error in the Central Auditory Nervous System (CANS). This means that if the brain attributes relevance to this signal, it can be perceived as a legitimate sensory stimulus rather than random noise, leading to its persistence.

Among the existing explanations, the chaos theory stands out, emphasizing the dynamic and nonlinear nature of brain activity. This theory emphasizes that small changes in the auditory pathway input can trigger neuroplastic reorganization and activate various neural networks, such as those involved in perception, salience, learning, and distress, resulting in the perception of tinnitus as bothersome, characterizing the symptom as tinnitus disorder^(3)^. In parallel, network theory suggests that the brain operates through interconnected nodes, simultaneously activating different circuits according to the stimuli and functions involved^(4) .^

Recent studies have identified several changes in the neural network of patients with tinnitus, covering the auditory, limbic, and attention systems, the default mode network, and areas related to memory, emotion, attention, and inhibitory control^(5)^. In addition, evidence suggests that the frontotemporal, parietofrontal, and temporo-parietal junctions of the left hemisphere play a fundamental role in the neural network of tinnitus, as they are linked to attention, auditory perception, memory^,^ and emotion^(6)^

To investigate the underlying mechanisms of the symptom and its relationship with neuroplasticity, researchers use Auditory Evoked Potentials (AEPs)^(7)^ . These potentials are neuroelectric recordings of the auditory pathway obtained through acoustic stimuli, enabling the analysis of neural activity and changes in the CANS. In addition, ABPs allow visualization of the activation of structures involved in the pathophysiology of tinnitus, reinforcing their relevance for assessment^(8) .^

Authors conducted a systematic review on the use of Brainstem Auditory Evoked Potentials (BAEP) to measure changes in the parameters analyzed when tinnitus is present. In specific analyses of interlatencies, the results show that ese tend to be increased, mainly in V-I, but with limited applicability, justified by sensorineural loss and not specifically by the presence of the symptom^(9)^. In this sense, Long Latency Auditory Evoked Potentials (LLAEP) are also used to perform this analysis, due to their relevant role in the analysis of patients with tinnitus disorder, mainly focused on the analysis of Cortical Auditory Evoked Potentials (CAEP).

AEPs are endogenous potentials that present automatic responses from the CANS. These are composed of the P1, N1, and P2 components, which represent the primary and secondary auditory cortex and reticular formation regions, responsible for perception^(10)^, decoding, discrimination, and auditory attention^(11)^. Accordingly, tinnitus can cause communication impairments^(12)^ and changes in the functioning of the frontal, parietal, and temporal regions, which are of paramount importance for proper auditory performance^(6)^. Thus, considering the neurophysiological changes caused by tinnitus and the regions analyzed by BAEPs, its clinical applicability stands out, as well as the gaps in knowledge about neural interconnections in the thalamocortical region, demonstrating the need for further investigation.

This raises the question of whether cortical potentials could be important diagnostic tools, considering that tinnitus can alter the functioning of the thalamocortical region and the primary/secondary auditory cortex, areas that are activated in this test. Thus, the present study is justified, seeking to broaden the understanding of these structures and evaluate the feasibility of this analysis in clinical practice. It is important to emphasize the importance of using verbal stimuli in BAEP, since tinnitus can influence speech perception and the brain areas involved in its generation, allowing for a more detailed representation of neural interconnections^(13)^.

The study hypothesis proposes that the presence of tinnitus disorder in adults is associated with significant changes in the components and, consequently, in the interlatencies of cortical potential waves, suggesting diffuse disorganization in several brain areas, especially in the thalamocortical regions and primary and secondary auditory cortex. As a consequence, there would be changes in neural processing and connectivity between auditory responses, objectively demonstrating the communicative behaviors faced in tinnitus disorder^(12)^.

Given this, considering the extensive brain changes observed in individuals with tinnitus, this study aims to compare response time and neural y between the cortical components of the BAEP, measuring latency and interpeaks in individuals with and without tinnitus

## METHOD

### Study design

This is an analytical, cross-sectional, quantitative study that was approved by the Research Ethics Committee under number 64696022.1.0000.5346. The study followed the rules and guidelines of Resolution 466/12 of the National Health Council, and all individuals who consented to participate in the research signed the Free and Informed Consent Form (FICF), which included a description of the procedures, risks, benefits, and confidentiality of the data.

### Participants

The sample was selected for convenience, and contact was made through social media posts by the audiology service at the location where the research was conducted. The procedures were performed at the teaching clinic of the institution of origin, from June 2023 to January 2024.

For both groups, the eligibility criteria were established as follows: individuals aged between 18 and 55 years, of both sexes, speakers of Brazilian Portuguese, right-handed, educated (more than twelve years of schooling), tonal hearing thresholds within normal standards, up to 19dBHL, at all conventionally assessed frequencies, without middle ear changes, with contralateral acoustic reflexes present at normal levels, normality on the Brief Neuropsychological Assessment Instrument-NEUPSILIN, functional integrity in the Brainstem Auditory Evoked Potential (BAEP)^(14)^, presence of all components of cortical potentials, and no negative self-perception regarding speech perception or changes in the battery of behavioral tests applied to assess Central Auditory Processing (CAP).

The exclusion criteria for both groups were: musicians or individuals exposed to musical practice who presented with cognitive complaints or diagnosed and/or evident neurological and/or psychiatric impairment, as well as dizziness or continuous exposure to noise or a history of traumatic brain injury.

For individuals with tinnitus disorder, the following eligibility criteria were added: perception of tinnitus in both ears, with a perception time greater than six months (confirming chronicity) and no evidence of a vascular component (pulsatile tinnitus). Furthermore, the perception of the symptom should cause complaints of impacts on quality of life, characterizing tinnitus disorder, with a score on the Visual Analog Scale (VAS) of at least 4 points and scores above 18 points on *the Tinnitus Handicap Inventory,* considering at least moderate discomfort and mild tinnitus. Those who were undergoing or had undergone another intervention for the symptom and/or who were using continuous medication or pharmacological treatment for tinnitus were excluded.

Eighty-five individuals of both sexes underwent evaluations, of whom 50 (58.82%) complained of tinnitus perception. Of the individuals with perception of the symptom, six (12%) were excluded due to middle ear changes, 10 (20%) due to neurological and/or psychiatric diseases, six (12%) because they were already undergoing treatment for tinnitus, and 12 (24%) because they had hearing loss. As for individuals without perception, 35 individuals (41.18%) were seen, of whom , 13 (37.14%) were excluded due to central auditory processing disorders and 10 (28.57%) due to a diagnosis of hearing loss.

Thus, according to the established eligibility criteria, the sample consisted of 28 participants, who were subdivided into two groups:

- **Control Group (CG):** composed of 12 individuals, three males and nine females, with a mean age of 23.89 years (standard deviation = 4.69 years) and 13.25 years of schooling (standard deviation = 2.70 years), without perception of tinnitus.
- **Study Group (SG):** composed of 16 participants, six males and 10 females, with a mean age of 35.75 years (standard deviation = 13.48 years) and 12.31 years of schooling (standard deviation = 1.54 years), with tinnitus disorder. Regarding the characteristics of tinnitus, a mean perception time of 4.95 years (minimum=1; maximum=10) was observed, with 13 having single perception (81.25%) and 3 having multiple perceptions (18.75%). Of these, eight had whistling tinnitus (50%), four had hissing tinnitus (25%), one had cricket-like tinnitus (6.25%), two had whistling and hissing tinnitus (12.5%), and one had cricket-like and hissing tinnitus (6.25%). Furthermore, for the VAS, a mean of 6.81 points (minimum=4; maximum=10) was observed, and for the THI, 51 points (minimum=20; maximum=94), demonstrating discomfort and a moderate degree of the symptom.

The variables gender (p-value=0.496), age (p-value=0.004), and education level (p-value=0.944) were analyzed between the groups to observe the homogeneity of the sample, which showed statistically significant differences only for age. It should be noted that the variables education and gender did not influence the comparisons, nor did age group, since one study did not show significant differences in the latency values of cortical AEPAs in the age group included^(15)^.

### Methodological design

This study was divided into two days. On the first day, the procedures for sample composition were performed: semi-structured questionnaire, basic audiological evaluation, application of behavioral tests of central auditory processing, neuropsychological evaluation, and self-perception questionnaires on tinnitus. On the second day, the BAEP-click was performed, despite this being a procedure for sample composition, and the BAEP-verbal (research procedures), due to the use of the same equipment and physical space for both measurements. Initially, the BAEP was performed with verbal stimulation, followed by the BAEP-neurodiagnostic. For both days, the total collection time was approximately one hour and 30 minutes, totaling three hours. It should be noted that the assessments were always performed in the same order in both groups.

The procedures for sample composition were performed to ensure the physical and functional integrity of the central auditory-cognitive structures, ensuring that changes in peripheral hearing acuity, acoustic signal processing, and/or reduction in cognitive aspects do not influence the BAEP components.

For a better methodological understanding, the procedures were divided into procedures for sample composition (audiological assessment, cognitive assessment, assessment of central auditory processing skills, and electrophysiological assessment - BAEP-neurodiagnosis) and research procedures (electrophysiological assessment - BAEP).

### Procedures for sample composition

#### Audiological assessment

- **Semi-structured questionnaire:** This was conducted to obtain information about the participants' identification, medical history, as well as aspects related to hearing and eligibility criteria. The questionnaire addressed hearing complaints, auditory processing, cognition, lifestyle habits, and past and current health conditions.
- **Visual inspection of the external auditory canal:** A *Mikatos* TK otoscope was used to check for possible changes that could interfere with the tests. In cases where any abnormality was detected, the participant was referred for medical evaluation.
- **Pure tone audiometry (PTA):** The test was performed in an acoustic booth with an AD229 audiometer (*Interacoustics*) and TDH 39 headphones, investigating air conduction hearing thresholds at frequencies from 250 to 8,000 Hz. Hearing thresholds up to 19 dBH, according to the criteria of the World Health Organization in the year 2021, available in the Audiological Assessment Guide of the Brazilian Federal Council of Speech-Language Pathology and Audiology ^(16)^. Were considered normal^.^The analysis was performed by isolated frequency, as even small changes can impact acoustic signal processing.
- **Audiometry:** Applied using the same equipment as for TPA, the assessment followed two stages. In the first stage, the Speech Recognition Threshold was determined by adding 30 dBHL to the tritonal average using the descending-ascending technique. The threshold was identified when the participant correctly repeated 50% of the four words presented. In the second stage, the Speech Recognition Percentage Index (SRPI) was assessed by adding 40 dBLH to the tritonal average or at a comfortable intensity. Twenty-five words were presented, each corresponding to 4% accuracy. Recognition was considered normal when the accuracy rate exceeded 90%, according to criteria already proposed^(16)^.
- **Acoustic immittance measurements:** The exam was performed with AT235 (Interacoustics) equipment and TDH-39 headphones, using a 226 Hz probe. Normal curves (type A) were considered those with a volume between 0.30 and 1.65 ml and pressure between 0 daPa and -100 daPa. Contralateral acoustic reflexes were evaluated at frequencies of 500, 1000, 2000, and 4000 Hz and were considered present and normal when triggered between 70 and 100 dB above the afferent airway threshold, according to criteria already proposed.^(16)^.

Exclusively for participants with tinnitus disorder, a specific medical history was taken, addressing general health history and factors that could influence the symptom. Through this procedure, a diagnosis of chronic bilateral tinnitus was reached, based on the individual's self-perception of the location and characteristics of the tinnitus. In addition, two instruments were applied: the Visual Analog Scale (VAS) and the Tinnitus Handicap Inventory (THI), aiming to measure the impacts and damages caused, as well as to identify the symptom as "Tinnitus Disorder."

The VAS was made available in printed format, numbered from 0 to 10, where one extreme indicated "absence of tinnitus" and the other represented the "worst tinnitus imaginable." During data collection, participants were instructed to indicate the level of discomfort perceived at the time of assessment. Moderate discomfort was considered when the score assigned was equal to or greater than 4 points^(17)^.

The THI questionnaire was administered to all participants who reported the presence of tinnitus, with the aim of assessing their quality of life and classifying it into different degrees according to the score obtained in the test. Comprising 25 questions, each answer received a specific score, and at the end of the questionnaire, the sum of the values determined the degree of impact of the symptom on the individual's well-being^(18)^.

#### Assessment of auditory skills

Behavioral tests to assess hearing skills were conducted in an acoustically treated booth using Telephonics TDH39 supra-aural headphones. These headphones were connected to a two-channel audiometer, model AD629B from Interacoustics, which was linked to a notebook computer to direct the assessments.

The assessments were performed at an intensity of 40 dBSL above the tritonal average^(19)^, applied alternately to minimize the effect of fatigue on participants. It should be noted that the use of this intensity is recommended by regulatory agencies and is also a strategy adopted by the percentage rate of speech recognition. However, in cases of individuals with reduced peripheral hearing acuity, this approach should be reevaluated. All tests were administered in a single session, with breaks for rest when necessary. Performance below expectations in at least one of the tests was considered indicative of Central Auditory Processing Disorder (CAPD).

To meet the minimum guidelines recommended by the American *Speech-Language-Hearing Association* (ASHA)^(20)^, the following tests were selected:

- **Dichotic Digits Test (DDT):** Assessed figure-ground auditory ability for verbal sounds in the binaural integration stage. The participant was asked to repeat the four numbers presented simultaneously, two in each ear, without following a specific order. The final percentage of correct answers per ear was obtained by subtracting the total number of errors multiplied by 2.5% from 100%. Results equal to or greater than 95% were considered normal^(21)^.
- **Pitch Pattern Sequence (PPS) - Auditec:** Aimed at assessing the temporal ordering of nonverbal sounds, this test required the participant to identify the sequence of three stimuli presented, classifying them as "thin" or "thick" (example: thin-thin-thick). Performance was considered normal when the accuracy rate was at least 86.6%^(22)^.^.^
- **Masking Level Difference (MLD):** Investigated binaural interaction. Participants were asked to answer "no" when they heard only noise or hissing and "yes" when they identified the whistle sound. The normality criterion established values equal to or greater than 8 dB^(22)^.
- **Speech in Noise Test (FR):** Assessed the ability to close out auditory interference for verbal sounds. Twenty-five monosyllabic words were presented to each ear, accompanied by ipsilateral white noise, with a signal-to-noise ratio (SNR) of 5 dB. The participant was instructed to ignore the noise and repeat the words heard. A minimum performance of 70% correct answers in both ears was considered normal^(21)^.
- **Gaps in Noise (GIN):** Applied to assess auditory temporal resolution. The participant was asked to raise their hand when they detected a silent interval between stimuli. The detection threshold was determined by the shortest interval correctly perceived in at least 4 of 6 presentations. Normal values were established at ≤ 5 ms. Only band 1 was used in both ears to optimize application time^(23)^.

#### Cognitive assessment

- **Brief Neuropsychological Assessment Instrument (NEUPSILIN):** NEUPSILIN was applied with the aim of drawing up a brief neuropsychological profile, both quantitative and qualitative, identifying possible preservation or impairment in the cognitive abilities of the participants. The instrument consists of 32 subtests aimed at assessing nine cognitive functions: Temporal-Spatial Orientation, Attention, Perception, Memory, Arithmetic Skills, Oral and Written Language, Praxias, and Executive Functions. In the present study, due to the influence of attentional and memory functions in eliciting the BAEP, only the subtasks related to these skills were applied and analyzed. The normality criteria adopted followed the normative parameters of Fonseca, Salles e Parente (2009)^(24) ,^taking into account the age and education level of the evaluated population.

#### Electrophysiological assessment of hearing

Before the start of the electrophysiological procedures, the electrode insertion areas were cleaned with abrasive paste. Then, disposable electrodes were fixed at specific points to ensure accuracy in the recordings. The evaluation was performed using *Smart* EP equipment from *Intelligent Hearing Systems* (IHS). The impedance of the electrodes was kept below 3 kΩ, while the difference between the electrodes remained below 2 kΩ. The transducer used was the ER-3A model, applied to both types of potentials. The electrophysiological evaluation included the following auditory potentials:

- **Brainstem Auditory Evoked Potential - Click (BAEP-Click):** This test aimed to verify the integrity of the auditory pathway at the brainstem level. The electrodes were positioned at points Fpz, Fz, A1, and A2. The stimulus used was a 100 ms click, in rarefied polarity, presented at an intensity of 80 dBHL. A total of 2,048 stimuli were applied, at a speed of 27.7 stimuli per second, with a gain of 100.0K and a bandpass filter between 100 and 3,000 Hz, with a recording window of 12 ms. Two acquisitions were performed to mark the waves, requiring replicability. The synchrony of the auditory pathway was considered within the normative standards when the latency values of waves I, III, and V, the interpeak intervals I-III, III-V, and I-V, in addition to the interaural difference of wave V and the ratio of waves V/I, were within the established references. The parameters and standards of normality followed the guidelines proposed by Webster^(14)^. ^,^adopting a criterion of two standard deviations. During the procedure, the participant remained relaxed and with their eyes closed.

#### Research procedure

- **Auditory Cortical Evoked Potential-verbal:** This test was conducted to investigate the neural activity of the central nervous system (CNS). The assessment was conducted binaurally, using earbuds and applied at an intensity of 80 dBHL. During the procedure, 300 verbal stimuli were presented, composed of the syllables /ba/ and /di/, which represented, respectively, the frequent stimulus (80% of presentations) and the rare stimulus (20% of presentations), following the oddball paradigm. Initially, a simulation of the test was performed, in which the evaluator orally emitted the sequence /ba/ and /di/, allowing participants to understand how the evaluation worked. Subsequently, the individuals were instructed to mentally count the occurrence of the /di/ stimulus. At the end of the exam, the examiner asked each participant to report the number of stimuli counted, comparing this value to the actual total of targets presented by the equipment. This procedure ensured that the task was performed correctly.

**Figure 1:**
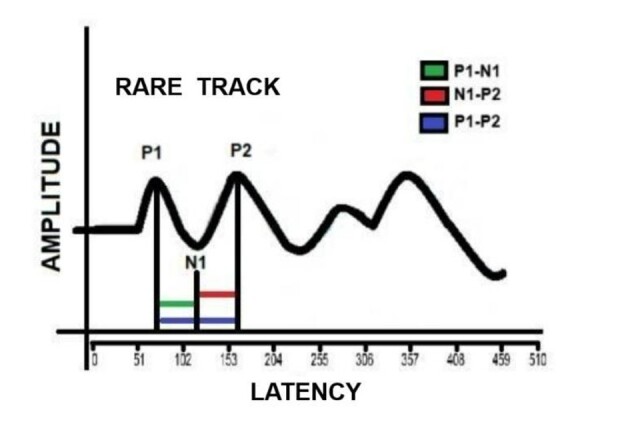
Graphical representation for marking the interpeaks of the cortical components of verbal BAEP

The parameters used in the assessment included a stimulation rate of 1.10 stimuli per second, a recording window of 510 ms, a gain of 100K, a filter of 100-3000 Hz, and an electroencephalogram window of 31%. The P1, N1, and P2 waves were marked exclusively on the rare trace. In addition, the interpeaks of the components were measured in ms, considering and performing the subtraction between the latency of the final component and the initial one (formula = N1 latency - P1 latency; P2 latency - N1 latency; P2-P1 latency) (Figure 1).

The reference values for latency and amplitude followed the criteria of Bruno; Oppitz; Garcia; Biaggio (2016)^(25)^, considering an interval of two standard deviations.

Figure 1 shows the graphical representation for marking the interpeaks of the verbal BEAP.

After acquisition, the tracings were sent without marking to two expert judges with expertise in verbal BAEP to perform their markings, with the tracings being considered only when there was 100% agreement. If there was no agreement, it was sent to a third expert judge to select the component marking location.

#### Data analysis

The data were entered into an *Excel* spreadsheet and statistical analyses were performed using SPSS *software*. Initially, the Shapiro-Wilk test was used to verify the normality of the data and, consequently, the choice of statistical test. The analysis between groups was performed using the T-test for independent samples. A significance level of 5% was considered for all analyses performed.

## RESULTS

A statistically significant difference was found in the comparison of the interpeak values of the P1-P2 potentials and a trend toward significance between N1-P2 in the comparison between the groups for the right ear and a trend toward significance in the comparison of the interpeak values of the P1-P2 potentials in the comparison between the groups for the left ear (Table 1).

**Table 1:** Comparison of PEAC-verbal cortical interpeak values between groups for right and left ears

**Interpeak RE**	**Group**	**n**	**Mean ± SD**	**Min - Max**	**P-value**
**P1-N1**	CG	12	44.17 ± 10.25	24.00 - 58.00	0.217
	SG	16	49.94 ± 13.05	29.00 - 77.00	
**N1-P2**	CG	12	66.58 ± 14.35	40.00 - 90.00	**0.075** *
	SG	16	79.56 ± 20.79	38.00 ± 108.00	
**P1-P2**	CG	12	110.75 ± 15.19	92.00 - 148.00	**0.025** **
	SG	16	129.00 ± 22.99	89.00 - 168.0	
**Interpeak LE**	**Group**	**n**	**Mean ± SD**	**Min - Max**	**P-value**
**P1-N1**	CG	12	45.25 ± 10.43	27.00 - 58.00	0.589
	SG	16	47.81 ± 13.43	27.00 - 65.00	
**N1-P2**	CG	12	65.08 ± 18.95	35.00 - 94.00	0.166
	SG	16	75.81 ± 20.26	45.00 - 114.00	
**P1-P2**	CG	12	110.33 ± 17.58	90.00 - 150.00	**0.090** *
	SG	16	123.63 ± 21.18	93.00 - 175.00	

* trend toward statistical significance;

** statistical significance; T-test for independent samples used

**Caption:** RE: right ear; LE: left ear; n: sample size; CG: control group; SG: study group

**Figure 2:**
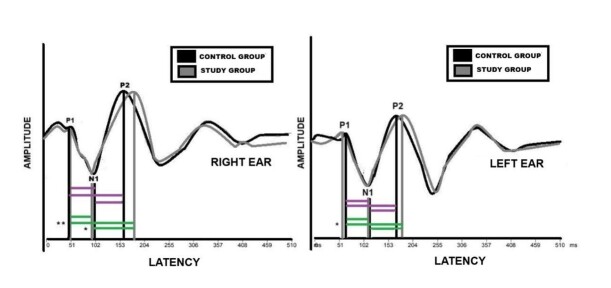
Graphical representation of the grand mean of the interpeaks of the P1-N1, P2-N2, and P1-P2 components of the verbal BAEP between groups for both ears

Figure 2 shows the graphical representation of the verbal BAEP interpeaks for the control and tinnitus groups in the right ear, from which it is possible to observe increased latencies for individuals with tinnitus disorder, with a trend toward significance for N1-P2 and statistical significance for P1-P2 (Figure 2).

**Caption:** purple line: interpeaks control group; green line: interpeaks study group; **: trend toward statistical significance; *: statistically significant difference

## DISCUSSION

This research is consistent with clinical tinnitus practice and the specialized literature, given that different perspectives and parameters related to the evaluation of verbal LAEP are currently cited, mainly measuring the presence or absence, as well as the latency and amplitude of the components of this potential^(7)^. In this sense, conducting new analyses for BAEP becomes of paramount importance for understanding diffuse changes and the neuroelectrical functioning of the central auditory pathway , since it reflects the functionality of the auditory pathway, which can represent with greater specificity the difficulties encountered in this population.

The analysis of inter-latencies in individuals with tinnitus was initially performed using BAEP-click^(26)^. A recent literature review showed that such analyses do not seem to present significant values in tinnitus, since in brainstem regions the changes are evidenced by increased central gain and not by changes in response time (interconnections)^(9)^. Thus, authors emphasize the need for further analysis, since the symptom may be related to decreased connectivity between the auditory cortex and the inferior colliculus, emphasizing that greater responses can be evidenced in higher evoked potentials^(27)^.

The BAEP is represented by the P1-N1-P2 wave complex, which reflects afferent thalamocortical activity (P1) and activity generated within, and connections between, the auditory cortex and non-auditory cortical regions (N1 and P2). A recent study analyzed the BAEP areas, demonstrating that when compared to their peers, they were increased, i.e., there is an increase in excitatory auditory cortical neural function and a reduction in inhibitory auditory cortical neural function, causing the perception of the symptom due to sensory *gating* alteration^(28)^. Thus, the analysis of inter-latencies seems to demonstrate the changes caused in this region, mainly in the interconnections between structures.

Table 1 shows significant differences for the P1-P2 interpeaks of the right ear and a tendency toward significance for N1-P2 of the same ear and P1-N1 of the left ear. It should be noted that the P1 component demonstrates sound perception, N1 demonstrates decoding, and P2 demonstrates auditory discrimination and attention. reference. Thus, the increase in inter-response latencies can be justified due to impairments related to such structures and functions, which may be altered in individuals with tinnitus disorder^(3,10)^. ^.^Although the present study did not find a statistically significant difference, a possible trend of variation between groups was observed, with increased values in patients with tinnitus disorder. The high variability and small sample size may have influenced the absence of statistical significance.

Figure 2 shows a graphical representation of the interpeak intervals, showing greater responses for the N1-P1 and P1-P2 components in individuals with tinnitus disorder. These findings can be justified because the cortical components of verbal BAEP reflect the neural functioning of the CANS in the thalamocortical region, primary and secondary auditory cortex, ^and^ reticular formation^(10)^. Thus, considering the auditory regions and functions that are measured by verbal BAEP, as well as a recent study that showed brain connections in the population that are mediated by cortico-cortical projections directly or through the thalamus^(29) ,^longer inter-structure response times are justified.

A study that sought to analyze neural changes in individuals with tinnitus demonstrated that the frontotemporal, parietofrontal, and temporo-parietal junctions of the left hemisphere are crucially involved in the symptom network^(6)^. These findings justify the greater changes observed in the right ear, given the left hemispheric dominance for processing verbal stimuli. Thus, acoustic processing aimed at decoding, discrimination, and auditory attention are delayed consistent^(13)^ with the greater responses in neural interconnections and, mainly, functional complaints^(12)^.

Due to the novelty of the study, which sought to analyze the interpeak intervals in verbal BAEP, no studies were found that could compare them to the research findings. However, the potential of the research is emphasized, so that it can be a new analysis employed in the potential, considering that the differences observed between the groups may evidence an increase in the responses of the central structures, reflecting the difficulties faced by individuals with tinnitus disorder, especially with regard to speech perception^(16)^. Therefore, despite the adequate response time, the disorganization in the functioning of structural interconnections seems to represent with greater reliability the neurophysiological changes faced in this population.

### Study limitations

The statistically significant differences in the interpeak values of cortical potentials (right ear: N1-P2 and left ear: P1-P2) may be related to the small sample size. The limitation in the number of participants is due to the strict exclusion criteria adopted, including age, education, cognition, central auditory processing, and hearing acuity, factors that could impact the results of the verbal CAEP. Further studies are needed to confirm these findings.

## CONCLUSION

Individuals with tinnitus disorder presented higher interpeak values in the BAEP, suggesting disorganization of central auditory functioning and, mainly, increased response between neural interconnections in acoustic processing.
