# Supplementary material for: Interpeak intervals of cortical components (P1-N1-P2): a comparative study between typical individuals and those with tinnitus disorder
Source: Codas. 2026 Jan 30;38(1):e20250130. doi: 10.1590/2317-1782/e20250130en (PMC12978340; doi:10.1590/2317-1782/e20250130en)
Supplement: Translation

# Translation: Interpicos dos componentes corticais (P1-N1-P2): um estudo de comparação entre indivíduos típicos e com transtorno do zumbido

**DOI:** 10.1590/2317-1782/e20250130pt

**Authors:** Hélinton Goulart Moreira, Christine Grellmann Schumacher, João Vitor de Aguiar Barcelos, Bruna Ribas Maia, Fabiana Cristina Toillier, Larissa Coradini, Isabela Pohlmann de Ávila Lourenço, Pedro Vinícius de Godoy Ferrão, Dayane Domeneghini Didoné, Michele Vargas Garcia

**Affiliations:** 1 Programa de Pós-graduação em Distúrbios da Comunicação Humana, Departamento de Fonoaudiologia, Universidade Federal de Santa Maria – UFSM - Santa Maria (RS), Brasil.; 2 Curso de Fonoaudiologia, Universidade Federal de Santa Maria – UFSM – Santa Maria (RS), Brasil.; 3 Universidade Federal de Santa Maria – UFSM - Santa Maria (RS), Brasil.; 4 Programa de Pós-graduação em Distúrbios da Comunicação Humana, Departamento de Fonoaudiologia, Universidade Federal de Santa Maria – UFSM - Santa Maria (RS), Brasil.

**Keywords:** Zumbido, Potenciais Evocados, Adultos, Sistema Nervoso Central, Córtex Auditivo

## Abstract

**Objetivo:**

Comparar tempo de resposta neural entre os componentes corticais do Potencial Evocado Auditivo de Longa Latência, medindo a latência e interpicos em indivíduos com e sem zumbido.

**Método:**

Estudo de caráter analítico, transversal e quantitativo. Participaram do estudo 28 indivíduos, divididos em dois grupos: Grupo Controle, composto por 12 indivíduos sem percepção do zumbido e Grupo Estudo, composto por 16 participantes com transtorno do zumbido. As avaliações foram divididas em dois dias. Inicialmente todos os pacientes foram submetidos a anamnese semi-estruturada, avaliação audiológica básica, testes comportamentais do processamento auditivo central, avaliação neuropsicológica e do zumbido. No segundo dia, foram realizados o PEAC-verbal e o PEATE-neurodiagnóstico. Foram comparadas as respostas da avaliação por meio da latência dos potenciais P1, N1, P2. A análise de comparação entre os grupos foi realizada por meio do teste T para amostras independentes.

**Resultados:**

Evidenciou-se diferença estatisticamente significante dos valores de interpicos dos potenciais P1-P2 e tendências à significância entre N1-P2 entre os grupos na orelha direita e na comparação de interpicos dos potenciais P1-P2 entre grupos na orelha esquerda.

**Conclusão:**

Indivíduos com transtorno do zumbido apresentaram interpicos maiores, o que sugere desorganização do funcionamento auditivo central e aumento de resposta entre as interconexões neurais no processamento acústico.

## INTRODUÇÃO

Inicialmente, a avaliação e o tratamento do zumbido concentravam-se na orelha interna, nos nervos cocleares e no sistema auditivo, buscando compreender sua fisiopatologia. Com o avanço das pesquisas, o modelo neurofisiológico emergiu como a principal explicação para a percepção e as manifestações desse sintoma. Esse modelo sugere que o zumbido decorre do aumento da atividade neural espontânea, mesmo sem um estímulo acústico específico, o que acaba ativando diferentes áreas cerebrais, com ênfase no sistema límbico^(1)^.

Nos últimos anos, as investigações científicas passaram a focar nas redes neurais associadas ao zumbido e em sua interação com outras regiões cerebrais e sistemas do organismo. Essa abordagem amplia a compreensão do sintoma além das suas causas primárias, explorando como diferentes áreas do cérebro contribuem para sua percepção. A teoria da codificação preditiva, proposta por Sedley et al.^(2)^, sugere que o zumbido pode ser interpretado como um erro de predição espontâneo no Sistema Nervoso Auditivo Central (SNAC). Isso significa que, caso o cérebro atribua relevância a esse sinal, ele pode ser percebido como um estímulo sensorial legítimo, em vez de um ruído aleatório, levando à sua persistência.

Entre as explicações existentes, destaca-se a teoria do caos, que enfatiza o caráter dinâmico e não linear da atividade cerebral. Nessa teoria, enfatiza-se que as pequenas alterações na entrada da via auditiva podem desencadear uma reorganização neuroplástica e ativar diversas redes neurais, como as de percepção, saliência, aprendizado e angústia, resultando na percepção do zumbido de forma incomodativa, caracterizando o sintoma como transtorno do zumbido^(3)^. Em paralelo, a teoria das redes sugere que o cérebro opera por meio de nodos interconectados, ativando simultaneamente diferentes circuitos conforme os estímulos e funções envolvidas^(4)^.

Estudos recentes identificaram diversas modificações na rede neural de pacientes com zumbido, abrangendo o sistema auditivo, límbico, de atenção, a rede de modo padrão e áreas relacionadas à memória, emoção, atenção e controle inibitório^(5)^. Além disso, evidências apontam que as junções frontotemporal, parietofrontal e tempo-parietal do hemisfério esquerdo exercem um papel fundamental na rede neural do zumbido, visto que estão ligadas à atenção, percepção auditiva, memória e emoção^(6)^.

Para investigar os mecanismos subjacentes do sintoma e sua relação com a neuroplasticidade, pesquisadores recorrem aos Potenciais Evocados Auditivos (PEAs)^(7)^. Estes potenciais são registros neuroelétricos da via auditiva obtidos por meio de estímulos acústicos, possibilitando a análise da atividade neural e das alterações no SNAC. Além disso, os PEAs permitem visualizar a ativação de estruturas envolvidas na fisiopatologia do zumbido, reforçando sua relevância para a avaliação^(8)^.

Autores realizaram uma revisão sistemática acerca do uso do Potencial Evocado Auditivo de Tronco Encefálico (PEATE) para mensurar as mudanças ocasionadas nos parâmetros analisados quando há a presença do zumbido. Nas análises específicas das interlatências, os resultados demonstram que essas tendem a ser aumentadas, principalmente, em V-I, no entanto com aplicabilidade limitada, justificadas pela perda sensorioneural e não especificamente pela presença do sintoma^(9)^. Nesse sentido, traz-se o Potencial Evocado Auditivo de Longa Latência (PEALL) para realização também desta análise, devido ao seu papel relevante na análise de pacientes com transtorno do zumbido, principalmente, voltado a análise dos Potenciais Evocados Auditivos Corticais (PEAC)

Os PEAC são potenciais endógenos, que apresentam respostas automáticas do SNAC. Esses são compostos pelos componentes P1, N1 e P2, que representam as regiões de córtex auditvo primário, secundário e formação reticular, responsáveis pelas habilidades de percepção^(10)^, decodificação, discriminação e atenção auditiva^(11)^. Em consonância, o zumbido pode ocasionar prejuízos comunicativos^(12)^ e mudanças de funcionamento em regiões de frontais, parietais e temporais de suma importância para o adequado desempenho auditivo^(6)^. Assim, tendo em vista as mudanças neurofisiológicas ocasionadas pelo zumbido e as regiões analisadas pelos PEACs, destaca-se a sua aplicabilidade clínica, bem como as lacunas no conhecimento sobre as interconexões neurais na região tálamo cortical, demonstrando a necessidade de maiores investigações.

Com isso, questiona-se se os potenciais corticais poderiam ser instrumentos diagnósticos importantes, considerando que o zumbido pode alterar o funcionamento da região tálamo-cortical e do córtex auditivo primário/secundário, áreas que são ativadas nesse exame. Dessa forma, justifica-se a realização da presente pesquisa, que busca ampliar a compreensão sobre essas estruturas e avaliar a viabilidade dessa análise na prática clínica. Ressalta-se, a importância do uso de estímulos verbais no PEAC, uma vez que o zumbido pode influenciar a percepção da fala e as áreas cerebrais envolvidas na sua geração, permitindo uma representação mais detalhada das interconexões neurais^(13)^.

A hipótese do estudo propõe que a presença do transtorno do zumbido em adultos esteja associada a alterações significativas nos componentes e, consequentemente, nas interlatências das ondas dos potenciais corticais, sugerindo uma desorganização difusa em diversas áreas cerebrais, sobretudo nas regiões tálamo-cortical e córtex auditivo primário e secundário. Como consequência, haveria mudanças no processamento neural e na conectividade entre as respostas auditivas, demonstrando objetivamente os comportamentos comunicativos enfrentados no transtorno do zumbido^(12)^.

Diante disso, considerando as alterações cerebrais amplas observadas em indivíduos com zumbido, esta pesquisa tem por objetivo comparar tempo de resposta neural entre os componentes corticais do PEAC, medindo a latência e interpicos em indivíduos com e sem zumbido

## MÉTODO

### Desenho do estudo

Trata-se de uma pesquisa de caráter analítico, transversal e quantitativo, que foi aprovada pelo Comitê de Ética em Pesquisa, sob número 64696022.1.0000.5346. O estudo seguiu as normas e diretrizes da Resolução 466/12 do Conselho Nacional de Saúde e todos os indivíduos que consentiram em participar da pesquisa assinaram o Termo de Consentimento Livre e Esclarecido (TCLE), em que constava a descrição dos procedimentos, riscos, benefícios e confidencialidade dos dados.

### Participantes

A amostra foi selecionada por conveniência, dos quais o contato foi realizado por meio de divulgação em redes sociais do serviço de audiologia no local em que a pesquisa foi realizada. Os procedimentos foram realizados na clínica escola da instituição de origem, no período de junho de 2023 a janeiro de 2024.

Para ambos os grupos, foram estabelecidos como critérios de elegibilidade: indivíduos com idades entre 18 e 55 anos, de ambos os sexos, falantes do português brasileiro, preferência manual direita, escolarizados (acima de doze anos de escolaridade), limiares auditivos tonais dentro dos padrões da normalidade, até 19dBNA, em todas as frequências convencionalmente avaliadas, sem alterações de orelha média, com reflexos acústicos contralaterais presentes em níveis normais, normalidade no Instrumento de Avaliação Neuropsicológica Breve-NEUPSILIN, integridade funcional no Potencial Evocado Auditivo de Tronco Encefálico (PEATE)^(14)^, presença de todos os componentes dos potenciais corticais e sem autopercepção negativa referente a percepção de fala ou alterações na bateria de testes comportamentais aplicada para avaliação do Processamento Auditivo Central (PAC).

Já os critérios de exclusão para ambos os grupos foram: indivíduos musicistas ou expostos à prática musical, que apresentassem queixas cognitivas ou comprometimento neurológico e/ou psiquiátrico diagnosticados e/ou evidentes, bem como de tontura ou exposição contínua ao ruído ou histórico de traumatismo cranioencefálico.

Para os indivíduos com transtorno do zumbido, foram acrescidos os seguintes critérios de elegibilidade: percepção de zumbido em ambas as orelhas, com tempo de percepção maior que seis meses (configurando cronicidade) e que não apresentassem indícios de componente vascular (zumbido do tipo pulsátil). Ainda, a percepção do sintoma deveria ocasionar queixas de impactos na qualidade de vida, caracterizando o transtorno do zumbido, com pontuação na Escala Visual Analógica (EVA) de no mínimo 4 pontos e escores superiores a 18 pontos no *Tinnitus Handicap Inventory*, considerando minimamente um incômodo moderado e um zumbido de grau leve. Foram excluídos aqueles que estavam ou realizaram outra intervenção para o sintoma e/ou que faziam o uso de medicação contínua ou tratamento farmacológico para o zumbido.

Foram submetidos às avaliações 85 indivíduos de ambos os sexos, dos quais 50 (58,82%) apresentavam queixa de percepção de zumbido. Dos indivíduos com percepção do sintoma, seis (12%) foram excluídos por alterações de orelha média, 10 (20%) por doenças neurológicas e/ou psiquiátricas, seis (12%) por já estarem realizando algum tratamento para o zumbido e 12 (24%) por terem perda auditiva. Já para os indivíduos sem percepção, foram atendidos 35 indivíduos (41,18%), dos quais 13 (37,14%) foram excluídos por apresentarem alteração de processamento auditivo central e 10 (28,57%) pelo diagnóstico de perda auditiva.

Desse modo, compuseram a casuística, de acordo com os critérios de elegibilidade estabelecidos, 28 participantes, dos quais foram subdivididos em dois grupos:

- **Grupo Controle (GC):** composto por 12 individuos, três do sexo masculino e nove do feminino, com média de idade de 23,89 anos (desvio-padrão= 4.69 anos) e 13,25 anos de escolaridade (desvio-padrão=2,70 anos), sem percepção de zumbido.
- **Grupo Estudo (GE):** Foi composto por 16 participantes, seis do sexo masculino e 10 do feminino, com média de idade de 35,75 anos (desvio-padrão= 13,48 anos) e 12,31 anos de escolaridade (desvio-padrão=1,54 anos), com transtorno do zumbido. Em relação às características do zumbido, observou-se uma média de tempo de percepção de 4,95 anos (mínimo=1; máximo=10), sendo 13 de percepção única (81,25%) e 3 com percepções múltiplas (18,75%). Desses, oito apresentaram zumbido do tipo apito (50%), quatro do tipo chiado (25%), um grilo (6,25%), dois apito e chiado (12,5%) e um grilo e chiado (6,25%). Ainda, para a EVA observou-se média de 6,81 pontos (mínima= 4; máxima= 10) e para o THI de 51 pontos (mínima= 20; máxima= 94), demonstrando incômodo e grau moderado para o sintoma.

Foram analisadas as variáveis sexo (p-valor=0,496), idade (p-valor=0,004) e escolaridade (p-valor=0,944) entre os grupos para observar a homogeneidade da amostra, das quais evidenciaram-se diferenças estatisticamente significantes apenas para a idade. Destaca-se que as variáveis escolaridade e sexo não influenciaram nas comparações, bem como a faixa etária, já que um estudo não evidenciou diferenças significantes nos valores de latência dos PEAs corticais na faixa etária incluída^(15)^.

### Delineamento metodológico

O presente estudo foi subdividido em dois dias, sendo no primeiro realizado os procedimentos para composição amostral: questionário semi-estruturado, avaliação audiológica básica, aplicação dos testes comportamentais do processamento auditivo central, avaliação neuropsicológica e questionários de auto percepção do zumbido. No segundo dia foi realizado o PEATE-clique apesar deste ser um procedimento para composição amostral e o PEAC-verbal (procedimentos de pesquisa), devido a utilização do mesmo equipamento e espaço físico para ambas mensurações. Inicialmente realizado o PEAC com estímulo verbal e, posteriormente, o PEATE-neurodiagnóstico. Para ambos os dias, o tempo total de coleta foi de aproximadamente uma hora e 30 minutos, contabilizando três horas ao total. Destaca-se que, as avaliações sempre foram realizadas na mesma ordem em ambos os grupos.

Os procedimentos para composição amostral foram realizados para garantir a integridade física e funcional das estruturas auditivas-cognitivas centrais, garantindo que alterações da acuidade auditiva periférica, processamento do sinal acústico e/ou redução dos aspectos cognitivos não ocasionem influência nos componentes do PEAC.

Para melhor compreensão metodológica, os procedimentos foram divididos em procedimentos para composição amostral (avaliação audiológica, avaliação cognitiva, avaliação do das habilidades do processamento auditivo central e eletrofisiológica - PEATE-neurodiagnóstico) e procedimentos de pesquisa (avaliação eletrofisiológica- PEAC)

### Procedimentos para composição amostral

#### Avaliação audiológica

- **Questionário semi-estruturado:** Foi conduzida com a finalidade de obter informações sobre a identificação dos participantes, histórico de doenças, além de aspectos relacionados à audição e critérios de elegibilidade. O questionário abordou queixas auditivas, processamento auditivo, cognição, hábitos de vida e condições de saúde passadas e atuais.
- **Inspeção visual do meato acústico externo:** Utilizou-se um otoscópio modelo TK, da marca *Mikatos*, para verificar possíveis alterações que pudessem interferir na realização dos exames. Nos casos em que alguma anormalidade fosse detectada, o participante foi encaminhado para avaliação médica.
- **Audiometria tonal liminar (ATL):** O exame foi realizado em cabina acústica com o audiômetro AD229 (*Interacoustics*) e fones TDH 39, investigando os limiares auditivos por via aérea nas frequências de 250 a 8.000 Hz. Consideraram-se dentro da normalidade os limiares auditivos até 19 dBNA, segundo os critérios da Organização Mundial da Saúde no ano de 2021, disponível no Guia de Avaliação Audiológica do Sistema de Conselhos de Fonoaudiologia^(16)^. A análise foi feita por frequência isolada, pois mesmo pequenas alterações podem impactar no processamento do sinal acústico.
- **Logoaudiometria:** Aplicada no mesmo equipamento da ATL, a avaliação seguiu duas etapas. Na primeira, foi determinado o Limiar de Reconhecimento de Fala, adicionando-se 30 dBNA à média tritonal, por meio da técnica descendente-ascendente. O limiar foi identificado quando o participante repetia corretamente 50% das quatro palavras apresentadas. Na segunda etapa, o Índice Percentual de Reconhecimento de Fala (IPRF) foi avaliado com acréscimo de 40 dBNA à média tritonal ou em intensidade confortável. Foram apresentadas 25 palavras, cada uma correspondendo a 4% de acerto. O reconhecimento foi considerado normal quando a taxa de acertos ultrapassou 90%, segundo critérios já propostos.
- **Medidas de imitância acústica:** O exame foi realizado com o equipamento AT235 (Interacoustics) e fones TDH-39, utilizando uma sonda de 226 Hz. Consideraram-se curvas normais (tipo A) aquelas que apresentavam volume entre 0,30 e 1,65 ml e pressão entre 0 daPa e -100 daPa. Os reflexos acústicos contralaterais foram avaliados nas frequências de 500, 1000, 2000 e 4000 Hz, sendo considerados presentes e normais quando ocorria o desencadeamento entre 70 e 100 dB acima do limiar da via aérea aferente, segundo critérios já propostos^(16)^.

Exclusivamente para os participantes com transtorno do zumbido, foi realizada uma anamnese específica, abordando o histórico geral de saúde e os fatores que poderiam influenciar o sintoma. Por meio deste procedimento, chegou-se ao diagnóstico de zumbido crônico bilateral, mediante autopercepção do indivíduo em relação à localização e características do zumbido. Além disso, foram aplicados dois instrumento: a Escala Visual Analógica (EVA) e o Tinnitus Handicap Inventory (THI), objetivando a mensuração dos impactos e prejuízos ocasionados, bem como a identificação do sintoma como “Transtorno do Zumbido”.

A EVA foi disponibilizada em formato impresso, numerada de 0 a 10, onde um extremo indicava "ausência de zumbido" e o outro representava o "pior zumbido imaginável". Durante a coleta de dados, os participantes foram instruídos a indicar o nível de incômodo percebido no momento da avaliação. Considerou-se incômodo moderado quando a pontuação atribuída era igual ou superior a 4 pontos^(17)^.

O questionário THI foi aplicado a todos os participantes que relataram a presença do zumbido, com o objetivo de avaliar sua qualidade de vida e classificá-la em diferentes graus conforme a pontuação obtida no teste. Composto por 25 perguntas, cada resposta recebeu uma pontuação específica, e ao final do questionário, a soma dos valores determinou o grau de impacto do sintoma no bem-estar do indivíduo^(18)^.

#### Avaliação das habilidades auditivas

Os testes comportamentais para avaliação das habilidades auditivas foram conduzidos em uma cabine com tratamento acústico, utilizando fones supra-aurais TDH39 da marca Telephonics. Esses fones estavam conectados a um audiômetro de dois canais, modelo AD629B da Interacoustics, que, por sua vez, estava vinculado a um notebook para direcionamento das avaliações.

As avaliações foram realizadas com uma intensidade de 40 dBNS acima da média tritonal^(19)^, aplicada de forma alternada para minimizar o efeito da fadiga nos participantes. Ressalta-se que o uso dessa intensidade é recomendado por órgãos regulamentadores, sendo uma estratégia adotada também no IPRF. No entanto, em casos de indivíduos com redução na acuidade auditiva periférica, essa abordagem deve ser reavaliada. Todos os testes foram aplicados em uma única sessão, com pausas para descanso quando necessário. O desempenho abaixo do esperado em pelo menos um dos testes foi considerado indicativo de Transtorno do Processamento Auditivo Central (TPAC).

Para atender às diretrizes mínimas recomendadas pela American *Speech-Language-Hearing Association* (ASHA)^(20)^, foram selecionados os seguintes testes:

- **Teste Dicótico de Dígitos (TDD):** Avaliou a habilidade auditiva de figura-fundo para sons verbais na etapa de integração binaural. O participante deveria repetir os quatro números apresentados simultaneamente, dois em cada orelha, sem necessidade de seguir uma ordem específica. O percentual final de acertos por orelha foi obtido subtraindo de 100% o total de erros multiplicado por 2,5%. Resultados iguais ou superiores a 95% foram considerados normais^(21)^.
- **Teste Padrão de Frequência (TPF) - Auditec:** Voltado para a avaliação da ordenação temporal de sons não verbais, esse teste exigiu que o participante identificasse a sequência de três estímulos apresentados, classificando-os como "fino" ou "grosso" (exemplo: fino-fino-grosso). O desempenho foi considerado dentro da normalidade quando a taxa de acertos foi de pelo menos 86,6%^(22)^.
- **Masking Level Difference (MLD):** Investigou a interação binaural. O participante deveria responder “não” ao perceber apenas ruído ou chiado e “sim” ao identificar o som do apito. O critério de normalidade estabeleceu valores iguais ou superiores a 8 dB^(22)^.
- **Teste de Fala no Ruído (FR):** Avaliou a habilidade de fechamento auditivo para sons verbais. Foram apresentadas 25 palavras monossílabas em cada orelha, acompanhadas de ruído branco ipsilateral, com uma relação sinal-ruído (S/R) de 5 dB. O participante foi instruído a ignorar o ruído e repetir as palavras ouvidas. Um desempenho mínimo de 70% de acertos em ambas as orelhas foi considerado dentro da normalidade^(21)^.
- **Gaps in Noise (GIN):** Aplicado para avaliar a resolução temporal auditiva. O participante deveria levantar a mão ao detectar um intervalo de silêncio entre os estímulos. O limiar de detecção foi determinado pelo menor intervalo percebido corretamente em pelo menos 4 de 6 apresentações. Os valores de normalidade foram estabelecidos em ≤ 5 ms. Apenas a faixa 1 foi utilizada em ambas as orelhas para otimizar o tempo de aplicação^(23)^.

#### Avaliação cognitiva

- **Instrumento de Avaliação Neuropsicológica Breve (NEUPSILIN):** O NEUPSILIN foi aplicado com o objetivo de traçar um perfil neuropsicológico breve, tanto quantitativo quanto qualitativo, identificando possíveis preservações ou prejuízos nas habilidades cognitivas dos participantes. O instrumento é composto por 32 subtestes voltados para a avaliação de nove funções cognitivas: Orientação Têmporo-Espacial, Atenção, Percepção, Memória, Habilidades Aritméticas, Linguagem Oral e Escrita, Praxias e Funções Executivas. No presente estudo, devido à influência das funções atencionais e de memória no eliciamento do PEAC, apenas as subtarefas relacionadas a essas habilidades foram aplicadas e analisadas. Os critérios de normalidade adotados seguiram os parâmetros normativos de Fonseca et al.^(24)^, levando em consideração a idade e a escolaridade da população avaliada.

#### Avaliação eletrofisiológica da audição

Antes do início dos procedimentos eletrofisiológicos, as áreas de inserção dos eletrodos foram higienizadas com pasta abrasiva. Em seguida, eletrodos descartáveis foram fixados em pontos específicos para garantir precisão nos registros. A avaliação foi realizada utilizando o equipamento *Smart* EP, da marca *Intelligent Hearing Systems* (IHS). A impedância dos eletrodos foi mantida abaixo de 3 kΩ, enquanto a diferença entre os eletrodos permaneceu inferior a 2 kΩ. O transdutor empregado foi o modelo ER-3A, aplicado em ambos os tipos de potenciais. A avaliação eletrofisiológica incluiu os seguintes potenciais auditivos:

- **Potencial Evocado Auditivo de Tronco Encefálico - Clique (PEATE-Clique):** Esse exame teve como objetivo verificar a integridade da via auditiva no nível do tronco encefálico. Os eletrodos foram posicionados nos pontos Fpz, Fz, A1 e A2. O estímulo utilizado foi um clique de 100 ms, em polaridade rarefeita, apresentado a uma intensidade de 80 dBNA. No total, foram aplicados 2.048 estímulos, com velocidade de 27,7 estímulos por segundo, ganho de 100,0K e filtro passa-banda entre 100 e 3.000 Hz, com uma janela de registro de 12 ms. Para marcação das ondas foram realizadas duas aquisições, necessitando replicabilidade. A sincronia da via auditiva foi considerada dentro dos padrões normativos quando os valores de latência das ondas I, III e V, os intervalos interpicos I-III, III-V e I-V, além da diferença interaural da onda V e a relação das ondas V/I, estiveram dentro das referências estabelecidas. Os parâmetros e padrões de normalidade seguiram as diretrizes propostas por Webster^(14)^, adotando um critério de dois desvios-padrão. Durante o procedimento, o participante permaneceu relaxado e com os olhos fechados.

#### Procedimento de pesquisa

- **Potencial Evocado Auditivo Cortical- verbal:** Esse exame foi conduzido para investigar a atividade neural do sistema nervoso central (SNC). A avaliação foi conduzida de maneira binaural, utilizando fones de inserção e aplicada a uma intensidade de 80 dBNA. Durante o procedimento, foram apresentados 300 estímulos verbais, compostos pelas sílabas /ba/ e /di/, que representaram, respectivamente, o estímulo frequente (80% das apresentações) e o estímulo raro (20% das apresentações), seguindo o paradigma oddball. Inicialmente, foi realizada uma simulação do teste, na qual o avaliador emitiu oralmente a sequência /ba/ e /di/, permitindo que os participantes compreendessem o funcionamento da avaliação. Posteriormente, os indivíduos foram instruídos a contar mentalmente a ocorrência do estímulo /di/. Ao término do exame, o examinador solicitou que cada participante relatasse o número de estímulos contados, comparando esse valor ao total real de alvos apresentados pelo equipamento. Esse procedimento garantiu que a tarefa fosse executada corretamente.

Os parâmetros utilizados na avaliação incluíram uma taxa de estimulação de 1,10 estímulo por segundo, janela de registro de 510 ms, ganho de 100K, filtro de 100-3000 Hz e janela do eletroencefalograma de 31%. A marcação das ondas P1, N1, P2 foi realizada exclusivamente no traçado raro. Além disso, os interpicos dos componentes foram mensurados em ms, considerando e realizando a subtração entre a latência do componente final ao inicial (formula= latência N1 - latência P1; latência P2- latência N1; latência P2-P1) (Figura 1).

**Figura 1:** Representação gráfica para marcação dos interpicos dos componentes corticais do PEAC-verbal

Os valores de referência para latência e amplitude seguiram os critérios de Bruno; Oppitz; Garcia; Biaggio (2016)^(25)^, considerando um intervalo de dois desvios-padrão.

Na Figura 1, encontra-se a representação gráfica para a marcação dos interpicos do PEAC-verbal.

Após aquisição, os traçados foram enviados sem marcação a dois juízes especialistas doutores e com expertise em PEAC-verbal, para realização das suas marcações, sendo considerados os traçados somente quando houvesse 100% de concordância. Caso não houvesse, este foi enviado a um terceiro juiz especialista, a fim de selecionar o local de marcação do componente.

#### Análise dos dados

Os dados foram alocados em planilha do *Excel* e as análises estatísticas foram realizadas no *software* SPSS. Inicialmente foi utilizado o teste Shapiro-Wilk para verificar a normalidade dos dados e, consequentemente, escolha do teste estatístico. A análise entre os grupos foi realizada por meio do teste T para amostras independentes. Considerou-se o nível de significância de 5% para todas as análises realizadas.

## RESULTADOS

Foi evidenciada diferença estatisticamente significante na comparação dos valores de interpicos dos potenciais P1-P2 e tendência a significância entre N1-P2 na comparação entre os grupos para orelha direita e uma tendência à significância na comparação dos valores de interpicos dos potenciais P1-P2 na comparação entre os grupos para orelha esquerda (tabela 1).

**Tabela 1:** Comparação dos valores de interpicos corticais do PEAC-verbal entre os grupos para orelha direita e esquerda

**Interpicos OD**	**Grupo**	**n**	**Média ± DP**	**Mín - Máx**	**P-valor**
**P1-N1**	GC	12	44,17 ± 10,25	24,00 - 58,00	0,217
	GE	16	49,94 ± 13,05	29,00 - 77,00	
**N1-P2**	GC	12	66,58 ± 14,35	40,00 - 90,00	**0,075** ^*^
	GE	16	79,56 ± 20,79	38,00 ± 108,00	
**P1-P2**	GC	12	110,75 ± 15,19	92,00 - 148,00	**0,025** ^**^
	GE	16	129,00 ± 22,99	89,00 - 168,0	
**Interpicos OE**	**Grupo**	**n**	**Média ± DP**	**Mín - Máx**	**P-valor**
**P1-N1**	GC	12	45,25 ± 10,43	27,00 - 58,00	0,589
	GE	16	47,81 ± 13,43	27,00 - 65,00	
**N1-P2**	GC	12	65,08 ± 18,95	35,00 - 94,00	
	GE	16	75,81 ± 20,26	45,00 - 114,00	0,166
**P1-P2**	GC	12	110,33 ± 17,58	90,00 - 150,00	
	GE	16	123,63 ± 21,18	93,00 - 175,00	0,090*

* tendência a significância estatística;

** significância estatística; Utilizado Teste T para amostras independentes **Legenda:** OD: orelha direita; OE: orelha esquerda; n: tamanho da casuística; GC: grupo controle; GE: grupo estudo

Na figura 2 encontra-se a representação gráfica dos interpicos do PEAC-verbal para os grupos controle e zumbido na orelha direita, do qual torna-se possível observar latências aumentadas para os indivíduos com transtorno do zumbido, com tendência a significância para N1-P2 e significância estatística para P1-P2 (figura 2).

**Figura 2:** Representação gráfica da grande média dos interpicos dos componentes P1-N1, P2-N2 e P1-P2 do PEAC-verbal entre os grupos para ambas as orelhas

**Legenda:** linha roxa: interpicos grupo controle; linha verde: interpicos grupo estudo; **: tendência a significância estatística; *: diferença estatisticamente significante

## DISCUSSÃO

A presente pesquisa está em consonância com a clínica do zumbido e a literatura especializada, tendo em vista que, atualmente, cita-se diferentes perspectivas e parâmetros relacionados à avaliação do PEAC-verbal, principalmente, mensurando a presença ou ausência, bem como a latência e amplitude dos componentes deste potencial^(7)^. Nesse sentido, realizar novas análises para o PEAC torna-se de suma importância para a compreensão das mudanças difusas e do funcionamento neuroelétrico da via auditiva central, já que este reflete a funcionalidade da via auditiva em que pode representar com maior especificidade as dificuldades encontradas nesta população.

A análise das inter-latências em indivíduos com zumbido, inicialmente, foi realizada no PEATE-clique^(26)^. Uma revisão de literatura recente demonstrou que tais análises parecem não apresentar valores significativos no zumbido, já que em regiões de tronco encefálico as alterações são evidenciadas pelo aumento do ganho central e não pelas mudanças entre o tempo de resposta (interconexões)^(9)^. Assim, autores enfatizam a necessidade de maiores análises, já que o sintoma pode estar relacionado a conectividade diminuída entre o córtex auditivo e o colículo inferior, enfatizando que as respostas maiores podem ser evidenciadas nos potenciais evocados mais elevados^(27)^.

O PEAC é representado pelo complexo de onda P1-N1-P2 que reflete a atividade talamocortical aferente (P1) e a atividade gerada dentro, e as conexões entre, o córtex auditivo e as regiões corticais não auditivas (N1 e P2). Uma pesquisa recente analisou as áreas do PEAC, demonstrando que ao compará-la com seus pares elas apresentaram-se aumentadas, ou seja, há o aumento da função neural cortical auditiva excitatória e redução da função neural cortical auditiva inibitória, ocasionando a percepção do sintoma pela alteração de *gating* sensorial^(28)^. Deste modo, a análise das inter-latências parecem demonstrar as mudanças ocasionadas nesta região, principalmente, nas interconexões entre as estruturas.

Na tabela 1, evidencia-se diferenças significantes para os interpicos P1-P2 da orelha direita e tendência a significância para N1-P2 da mesma orelha e P1-N1 da orelha esquerda. Destaca-se que o componente P1 demonstra a percepção do som, o N1 a decodificação e o P2 a discriminação e atenção auditiva. referencia. Assim, o aumento de latências inter-repostas podem ser justificados devido aos prejuízos relacionados a tais estruturas e funções, que podem encontrar-se alteradas nos indivíduos com transtorno do zumbido^(3,10)^. Embora o presente estudo não tenha encontrado diferença estatisticamente significativa, observou-se uma possível tendência de variação entre os grupos, com valores aumentados nos pacientes com transtorno do zumbido. A alta variabilidade e o pequeno tamanho da amostra podem ter influenciado a ausência de significância estatística.

Na Figura 2, observa-se a representação gráfica dos intervalos interpicos, evidenciando respostas maiores para os componentes N1-P1 e P1-P2 nos indivíduos com transtorno do zumbido. Esses achados podem ser justificados devido aos componentes corticais do PEAC-verbal refletirem o funcionamento neural do SNAC, na região tálamo-cortical, córtex auditivo primário e secundário e formação reticular^(10)^. Desse modo, tendo em vista as regiões e funções auditivas que são mensuradas pelo PEAC-verbal, bem como um estudo recente que evidenciou as conexões cerebrais na população, que são mediadas por projeções córtico-corticais diretamente ou através do tálamo^(29)^, justificam os tempos de respostas inter-estruturas maiores.

Uma pesquisa que buscou analisar as mudanças neurais em indivíduos com zumbido, demonstrou que as junções frontotemporal, parietofrontal e tempo-parietal do hemisfério esquerdo estão crucialmente envolvidas na rede do sintoma^(6)^. Estes achados, justificam as maiores mudanças evidenciadas para orelha direita, tendo em vista a dominância hemisférica esquerda para o processamento de estímulos verbais. Assim, o processamento acústico voltado a decodificação, discriminação e atenção auditiva encontram-se defasados^(13)^, indo ao encontro das maiores respostas nas interconexoes neurais e, principalmente, as queixas funcionais^(12)^.

Devido ao ineditismo do trabalho, em buscar analisar os intervalos interpicos no PEAC-verbal, não foram encontrados estudos que pudessem compará-los aos achados da pesquisa. No entanto, salienta-se a potencialidade da pesquisa, para que esta possa ser uma nova análise empregada no potencial, tendo em vista que as diferenças observadas entre os grupos podem evidenciar o aumento nas respostas das estruturas centrais, refletindo as dificuldades enfrentadas por indivíduos com transtorno do zumbido, especialmente no que se refere à percepção da fala^(11)^. Portanto, apesar do tempo de respostas adequado, a desorganização no funcionamento das interconexões estruturais parece representar com maior fidedignidade as mudanças neurofisiológicas enfrentadas nesta população.

### Limitações do estudo

As tendências as diferenças estatisticamente significantes nos interpicos dos potenciais corticais (orelha direita: N1-P2 e orelha esquerda: P1-P2), podem estar relacionadas ao tamanho reduzido da amostra. A limitação no número de participantes deve-se aos critérios rigorosos de exclusão adotados, incluindo idade, escolaridade, cognição, processamento auditivo central e acuidade auditiva, fatores que poderiam impactar os resultados do PEAC-verbal. Salienta-se a necessidade de maiores estudos para ratificar os achados encontrados.

## CONCLUSÃO

Indivíduos com transtorno do zumbido apresentaram interpicos maiores no PEAC o que sugere desorganização do funcionamento auditivo central e, principalmente, aumento de resposta entre as interconexões neurais no processamento acústico.
